# Aqueous Extract of *Dendropanax morbiferus* Leaves Effectively Alleviated Neuroinflammation and Behavioral Impediments in MPTP-Induced Parkinson's Mouse Model

**DOI:** 10.1155/2018/3175214

**Published:** 2018-04-15

**Authors:** Shin-Young Park, Govindarajan Karthivashan, Hyun Myung Ko, Duk-Yeon Cho, Joonsoo Kim, Dae Jun Cho, Palanivel Ganesan, In Su-Kim, Dong-Kug Choi

**Affiliations:** ^1^Department of Applied Life Sciences, Graduate school of Konkuk University, Chungju 27478, Republic of Korea; ^2^Department of Biotechnology, College of Biomedical and Health Science, Konkuk University, Chungju 27478, Republic of Korea; ^3^Department of Eco-Biological Science, College of Science and Technology, Woosuk University, 66 Daehak-ro, Jincheon-eup, Chungcheongbuk-do 27841, Republic of Korea; ^4^Department of Quality Control, Namyang F&B Co. Ltd., Eumseong, Republic of Korea; ^5^Nanotechnology Research Center and Department of Applied Life Science, College of Biomedical and Health Science, Konkuk University, Chungju 27478, Republic of Korea

## Abstract

Parkinson's disease (PD) is a commonly reported age-related neurodegenerative disorder. Microglial-mediated neuroinflammation is one of the cardinal hallmarks of various neurodegenerative disorders, including PD progression. Inadequate therapeutic strategies and substantial adverse effects of well-established drug candidates demand new therapeutic leads to treat PD. *Dendropanax morbifera* (DM) is an endemic plant species of South Korea, and it has been used extensively as traditional medicine to treat numerous clinical complications. In this study, we conducted an initial profiling of the few major phytoconstituents of aqueous DM leaf extracts (DML) and quantified the same using high-performance liquid chromatography tandem mass spectrometry with electrospray ionization (HPLC-ESI-MS/MS). We subsequently evaluated the antineuroinflammatory activity and ameliorative potential of DML in both *in vitro* and *in vivo* experimental PD models. The prophylactic treatment of DML effectually improved the behavioral deficits, curbed the microglial-mediated neuroinflammation, and protected dopaminergic (DA) neuronal loss by restoring tyrosine hydroxylase (TH) levels in brain tissue of the MPTP-induced PD mouse model. We conducted chromatographic profiling and identified chlorogenic acid (CA) as a major constituent (19.5 mg/g of BuOH fraction), which has been well documented as an antioxidant and anti-inflammatory agent. This was found to be in harmony with our *in vitro* results, where DML suppressed the level of inflammatory mediators and allied the signaling pathway in LPS-stimulated microglial cells. The results of our study indicate that DML and its bioactive constituents can be developed as potential therapeutic candidates against progressive PD complications.

## 1. Introduction

Parkinson's disease (PD) is the second most commonly reported age-related neurodegenerative disorder, and it is substantially characterized by progressive dopaminergic (DA) neuronal loss in the substantia nigra pars compacta (SNpc) of the nigrostriatal system, functional impairment of microglial cells, and motor dysfunction (i.e., resting tremor, bradykinesia, and postural instability) [1, 2]. Microglia, the resident macrophages of the central nervous system (CNS), play a major role in retaining the homeostasis in the brain milieu by providing a first-line defense against various exogenous and endogenous contaminants, curbing the potential spread of injury inflicted by clinical complications [3]. Previous studies suggested that the microglia involved in the phagocytosing of dead neuronal cells in the brain milieu also aid in the survival of nerve cells by releasing varied neurotrophic factors [4, 5]. Increasing evidence suggests that the activation of resting glial cells was attained either by direct factors, that is, toxins, pathogens, or endogenous proteins, or by indirect factors due to neuronal death cascades [6, 7]. Precisely, activated glial cells trigger the inflammatory cascade by releasing proinflammatory cytokines (tumor necrosis factor-alpha (TNF-*α*) and interleukin (IL-1*β* and IL-6)) and inflammatory mediators (nitric oxide (NO), inducible nitric oxide synthase (iNOS), and cyclooxygenase-2 (COX-2)) by activating nuclear factor kappa-light-chain-enhancer (NF-*κ*B) and mitogen-activated protein kinase (MAPK) pathways [8, 9]. These cascades lead to NO accumulation and exhaustive DA neuronal loss, as clinically observed in the autopsy reports of several PD patients [10–13]. The exogenous activation of microglial cells was achieved both *in vitro* and *in vivo* via exposure of lipopolysaccharide (LPS), 1-methyl-4-phenyl-1,2,3,6-tetrahydropyridine (MPTP), and other neurotoxins [14–16]. In particular, the MPTP-induced mouse model is the most commonly used PD model, and it depicts significant clinical hallmarks of PD pathogenesis, that is, neuroinflammation and DA neuronal cell death more proximal to human brains [17]. MPTP was also reported to reduce the ratio of tyrosine hydroxylase (TH) positive in the SNpc and striatum (STR) regions compared to wild-type control, which subsequently contributes to the striatal DA deficiency and results in behavioral defects. In addition to the histopathological alterations, MPTP-treated mice exhibit substantial motor deficits, which shall be associated to tremors, rigidity, and posture imbalance of mice. However, experimentally, the behavioral alternation of the animals was measured using pole and rotarod tests, which shall relatively measure the motor skills of the animals (i.e., bradykinesia and hypokinesia). Current therapeutic strategies for PD provide inadequate benefits with substantial adverse effects; so to overcome this limitation, a safe and effective alternative should be developed. In recent years, phytotherapeutic leads have been in the limelight due to their improved potential with minimal or null toxicity in PD.

*Dendropanax morbiferus* (DM) is an endemic plant species that is widely distributed in the southern parts of Korea [18]. The leaves of this plant were used as prebiotic, probiotic, and antibacterial agents against various pathogens [19]. Various other parts of the plant have been documented as alternative, folkloric medicine against dermal complications and other infectious diseases [18–20]. Numerous scientific studies have reported the antioxidant, antidiabetic, anticarcinogenic, and nephroprotective potential of this plant species [21–23]. DM has also been recently reported to effectively alleviate the hippocampal function in mercury-induced neurotoxic rats by improving the endogenous antioxidant levels [24]. Though several studies reported broad medicinal properties of DML, its mechanism of action in the PD model has been poorly understood. In this study, we evaluated the underlying molecular mechanism of the antineuroinflammatory activity and the neuroprotective potential of DML and its bioactive compound (CA) in *in vitro* and *in vivo* experimental models of PD.

## 2. Materials and Methods

### 2.1. Reagents

*Dendropanax morbiferus* leaves were procured from Hanna arboretum (Jeonnam, Korea). 1-Methyl-4-phenyl-1,2,3,6-tetrahydropyridine (MPTP), lipopolysaccharide (LPS), dimethyl sulfoxide (DMSO), 3-(3,4-dimethylthiazole-2-yl)-2,5-diphenyl-tetrazoliumbromide (MTT), chloroform, quercetin, kaempferol, rutin, vitexin, luteolin, tricin, ferulic acid, caffeic acid, chlorogenic acid, and N-1(1-naphthyl)ethylene-diamine dihydrochloride were obtained from Sigma-Aldrich (St. Louis, MO, USA). A 10x RIPA buffer was obtained from Millipore (Milford, MA, USA). Protease inhibitor and phosphatase inhibitor cocktail tablets were purchased from Roche (Indianapolis, IN, USA). Plastic wares (6-well, 12-well, and 24-well tissue culture plates and 100 mm culture dishes) were purchased from SPL (Korea). Dulbecco's modified Eagle's medium (DMEM), fetal bovine serum (FBS), 1x trypsin-EDTA (TE), and 100,000 U/ml penicillin-streptomycin (P-S) were obtained from Gibco/Invitrogen (Carlsbad, CA, USA). Primary antibodies anti-ERK (1 : 2000), anti-p-ERK (1 : 2000), anti-JNK (1 : 1000), anti-p-JNK (1 : 1000), anti-p38 (1 : 2000), anti-p-p38 (1 : 2000), and anti-TH (1 : 1000) were obtained from Cell Signaling Technology (Danvers, MA, USA); anti-COX-2 (1 : 1000) and anti-TH (1 : 200) for IHC were obtained from Abcam (Cambridge, UK) and Calbiochem (San Diego, CA, USA); anti-Iba-1 (1 : 1000) and anti-iNOS (1 : 1000) were procured from Wako-chem (Chuo-ku, Osaka, Japan) and BD Biosciences (San Jose, CA, USA), respectively. Secondary antibodies antimouse (1 : 2000) and antirabbit (1 : 2000) were obtained from Cell Signaling Technology (Danvers, MA, USA) and Bio-Rad (Hercules, CA, USA), respectively.

### 2.2. Plant Leaf Extraction of *Dendropanax morbiferus* Leaves

DM leaves were purchased from Hanna arboretum, Republic of Korea. The obtained fresh DM leaves were washed in running tap water, oven-dried (50–60°C) for 3–5 days, and it is crushed manually to obtain the leaf flakes. The flakes were subjected to heat maceration in 1 l of distilled water at 100°C for 2 h, and the extract was filtered through Whatman® filter paper number 2. The obtained filtrate was further concentrated using rotary evaporator (EYELA N-1000, Tokyo), for 2 h, 3 times. The DM leaf extract (DML) residual was freeze-dried for 7 days and stored in an airtight container at −20°C.

### 2.3. Cell Culture Treatment

The BV-2 microglial cells were generously provided by Dr. K. Suk (Kyung-Pook National University, Daegu, Korea). As previously reported [25], the cells were cultured and maintained in Dulbecco's modified Eagle's medium (DMEM) supplemented with 5% FBS and 50 *μ*g/ml penicillin-streptomycin and maintained in a humidified incubator supplied with 5% CO_2_ and 95% O_2_. The cells were seeded at a density of 5 × 10^4^ cells/ml and were pretreated for 1 h with varying concentrations of DML (100, 250, and 500 *μ*g/ml), followed by LPS incubation (200 ng/ml) at the respective time points (30 min, 6 h, and 24 h).

### 2.4. Animal Experimental Design

Six-week-old male C57BL/6N mice were obtained from DBL (Chungbuk, Korea) and acclimatized for 1 week before the start of the experiment. The 7-8-week-old (25–27 g) mice were used in this study. All experiments were performed in accordance with the principles of laboratory animal care (NIH publication number 85-23, revised 1985) and were approved by Konkuk University Institutional Animal Care and Use Committee (KU 17009). The animals were housed in a controlled environment (23 ± 1°C and 50% ± 5% humidity; 12 h dark-light cycle) and allowed food and water ad libitum. The animals (*n* = 24) were divided into three groups (*n* = 8 per group). The groups included a vehicle group (untreated), MPTP group (20 mg/kg of bw—four times, day 7 at 2 h intervals, i.p.), and DML group (200 mg/kg of bw—single dose/day until day 7, p.o.). MPTP and DML were dissolved in saline and prepared just prior to dosing.

### 2.5. Cell Cytotoxicity and NO Release

BV-2 cells were seeded at a density of 5 × 10^4^ cells/well and were pretreated with various concentrations of DM (100, 250, and 500 *μ*g/ml) for 1 h, followed by LPS (200 ng/ml) induction for 24 h. 20 *μ*l of MTT (2.0 mg/ml) was added to each well, and after 2 h of incubation at 37°C in 5% CO_2_, the supernatants were removed from each well, and the formed formazan crystals in viable cells were dissolved in DMSO. The absorbance was determined at 540 nm using a microplate reader (Tecan Trading AG, Basel, Switzerland). The inhibitory effect of DM on NO production was determined as previously described [25]. BV-2 microglial cells (5 × 10^4^ cells/well) were incubated with LPS (200 ng/ml) in the presence or absence of DM (100, 250, and 500 *μ*g/ml) for 24 h. After 24 h, 100 *μ*l of supernatants was initially collected and assayed for NO release using commercially available Griess reagent (1 vol. 0.1% naphthylethylenediamine and 1 vol. 1% sulfanilamide in 5% H_3_PO_4_). Absorbance was determined at 540 nm using a microplate reader (Tecan Trading AG).

### 2.6. RNA Isolation and Reverse Transcription Polymerase Chain Reaction (RT-PCR) Analysis

Total RNA was isolated from BV-2 microglial cells (5 × 10^4^ cells/well) treated for 6 h, using TRIzol reagent (Invitrogen Life Technologies, Carlsbad, CA, USA) according to the manufacturer's instructions. cDNA synthesis was primarily performed using 2500 ng of total RNA and GoScript^TM^ Reverse Transcription System (Promega, USA). Polymerase chain reaction was performed using an initial step of denaturation (2 min at 95°C), 24–30 cycles of amplification (95°C for 30 s, 55–58°C for 45 s, and 72°C for 1 min), and extension at 72°C for 5 min. The primer sequences used for inflammatory genes are represented in Table 1. PCR products were analyzed in 1% agarose gels. For quantification, the gels were photographed and the pixel intensity for each band was determined using the ImageJ (NIH) software and was normalized to the band intensity of GAPDH mRNA. The results are representative of three independent experiments.

### 2.7. Quantitative Real-Time Polymerase Chain Reaction (qRT-PCR) Analysis

Real-time PCRs (RT-PCRs) were performed in a Roche LightCycler 96 Real-Time System using Power SYBR green master mix (Life Technologies), according to the manufacturer's instructions, in a final volume of 20 *μ*l reactions. The PCR conditions were as follows: initially incubated for 10 minutes at 95°C, followed by 15 sec incubation at 95°C (40 cycles) and a final 60 sec incubation at 60°C. Further, the specificity of each primer was verified by melting curve analysis (at 65–95°C, with fluorescence recording at every 0.5°C).

### 2.8. Immunocytochemistry (ICC) Analysis

BV-2 microglia cells (1 × 10^5^ cells/well in a 12-well plate) were cultured on sterile 12 mm cover slips in 24-well plates and treated with DM (100, 250, and 500 *μ*g/ml) and LPS (200 ng/ml) to detect the intracellular location of the nuclear factor kappa-B (NF-*κ*B) p65 subunit. A fluorescence immunocytochemistry assay was performed, and the representative images were obtained using a fluorescence microscope (Carl Zeiss Inc., Oberkochen, Germany) as previously described [25, 26].

### 2.9. Behavioral Studies

#### 2.9.1. Pole Test

The pole test for bradykinesia was conducted as previously described [27]. The mice were placed at the top of a rough-surfaced pole (8 mm diameter and 55 height) with the head-up posture, and the total locomotor activity (TLA) was measured. The TLA is the time taken by the mouse to reach the floor. The duration of these parameters reflects bradykinesia in PD. This test was performed successively five times for each mouse, and the average was analyzed.

#### 2.9.2. Rotarod Test

The rotarod test was conducted to evaluate the motor deficits, as previously described with slight modification [28]. A computerized, automated rotarod 5-unit lane (DBL-02-MA5, Korea) machine was used for each mouse per lane. The rotarod machine was set with a preprogrammed protocol and was allowed to rotate at selective speed and time limits. The machine detects the mouse fall and records the time and distance covered by each mouse on their corresponding lanes. The mice from all groups were pretrained in the rotarod machine, at 10 rpm (5 min), once per day for three consecutive days (days 5–7), prior to MPTP injection. On day 14, the experiment was started at a low speed limit of 4 rpm, with a gradual increase until reaching a high speed limit of 40 rpm. The time taken to fall—latency of fall (LTF) and total distance (TD) covered by each mouse during their respective sessions—was recorded and analyzed.

### 2.10. Western Blot Analysis

Treated BV-2 cells (5 × 10^5^ cells/well) and animal brain tissue were washed twice with PBS and lysed for 10 min using 1x RIPA lysis buffer (4°C), respectively. Cell and tissue lysates were centrifuged at 14,000 rpm, 4°C, and the corresponding supernatants were collected and separately stored for further analysis. The protein concentration of each sample was obtained using a DC Protein Assay kit (Bio-Rad). Equal amounts of protein (20–40 *μ*g for cells/600 *μ*g for animal) were separated electrophoretically by 10% sodium dodecyl sulfate-polyacrylamide electrophoresis, and the resolved proteins were transferred to polyvinylidene difluoride membranes (Millipore, Bedford, MA, USA). The membranes were incubated for 1 h with 3% BSA in TBS buffer to block nonspecific binding. The membranes were then incubated with primary antibodies to anti-inducible nitric oxide synthase, antityrosine hydroxylase (TH), anti-I*κ*B-*α*, anti-phospho-I*κ*B-*α*, anti-p65, anti-cyclooxygenase-2, anti-*β*-actin, and anti-ionized calcium-binding adapter molecule 1 (Iba-1), followed by incubation for 1 h with horseradish peroxidase-conjugated-specific secondary antibodies (1 : 2000; Cell Signaling, MA). The blots were visualized by a PowerOpti-ECL kit obtained from the Animal Genetics Inc. (Gyeonggi-do, Korea) detection system according to the recommended procedures.

### 2.11. Immunohistochemistry (IHC) Analysis

Following the behavioral tests, the mice were anesthetized using 23% urethane (i.p.) for immunohistochemical investigations. The brains of each mice were perfusion fixed via cardiac puncture with 4% paraformaldehyde, followed by a saline flush (Biosesang, Korea). The brains were removed after perfusion fixation at 4°C, immersed in the same fixative, and dehydrated in 30% sucrose solution. Subsequently, the brain was frozen-embedded using tissue freezing medium (Leica, GmbH Heidelberger, Germany). The fixed frozen brains were sectioned (30 *μ*m) coronally to obtain the striatum and substantia nigra (*n* = 3/group). The free-floating brain sections (30 *μ*m) were then incubated with specific anti-TH (1 : 200; Calbiochem; Merck KGaA, Darmstadt, Germany), VECTASTAIN ABC kit, and biotinylated secondary antibodies. The samples were visualized using DAB peroxidase (HRP) substrate kit (Vector Laboratories, CA, USA) by following the manufacturer's protocol.

### 2.12. Fractionation and Chromatographic Analysis

To identify the potential bioactive compounds responsible for the therapeutic efficacy of DML, we further obtained phenol-enriched ethyl acetate and butanol fractions of DML. The HPLC analysis of DML and its fractions were conducted using Agilent Technologies 6410 Triple Quad LC-MS/MS (Agilent, Santa Clara, CA, USA) with C18, 2.1 × 100 mm, 2.7 *μ*m column. The mobile phases—solvent A (0.1% formic acid in water) and solvent B (0.1% formic acid in acetonitrile)—were used at a flow rate of 400 *μ*l/min. The sample injection volume was 3 *μ*l/sample. The gradient program set for the analysis is as follows: 95% solvent A : 5% solvent B, 0–30 min; 50% solvent A : 50% solvent B, 30–40 min. Compound identification was achieved using a coupled mass spectrometry system with the following source parameters: gas temp 350°C; capillary volt. 4000 V; nebulizer 40 psi; fragmentor 190 V allied with a MassHunter Software system. To quantify the identified compounds, commercially acquired authentic standards of quercetin, kaempferol, rutin, vitexin, luteolin, tricin, ferulic acid, chlorogenic acid, and caffeic acid dissolved in MeOH were used for the analysis.

### 2.13. Statistical Analysis

All the data were analyzed using GraphPad Prism software ver. 5.01 (GraphPad Inc., La Jolla, CA, USA). All data are expressed as mean ± standard deviation of at least three independent experiments. The statistical analysis was performed with a one-way analysis of variance (ANOVA) followed by Tukey's multiple comparison tests. The *P* values < 0.05 were considered to be significant.

## 3. Results

### 3.1. Effects of DML on the Cell Viability and Nitric Oxide Production in LPS-Stimulated BV-2 Microglial Cells

**Figure 1:**
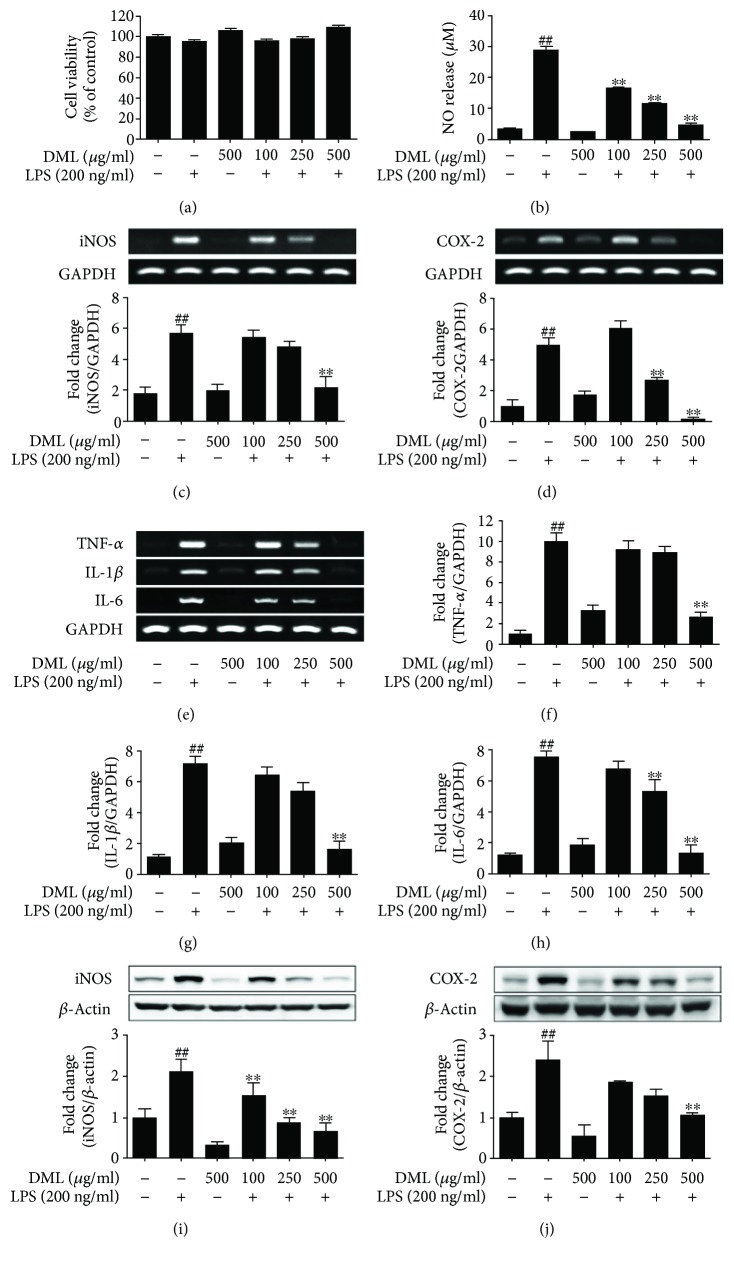
DML attenuates nitric oxide production, cell viability, and proinflammatory mediators in LPS-stimulated BV-2 cells. The cells were incubated with DML in the presence or absence of LPS (200 ng/ml) for 6 h (RNA levels) and 24 h (NO assay, MTT assay, and protein levels). The cytotoxicity and NO release results were displayed as percentage of control (a) and released NO in *μ*M (b), respectively. The RT-PCR results of the inflammatory cytokines were expressed as bands and fold-change quantification with respect to GAPDH ratio of iNOS (c), COX-2 (d), TNF-*α*, IL-1*β*, and IL-6 (e–h). The immunoblot results of inflammatory mediators iNOS (i) and COX-2 (j) were expressed as blots and fold-change quantification with respect to *β*-actin ratio. Data are presented as mean ± standard error (*n* = 3) of three independent experiments. The values are mean ± standard error (^##^*P* < 0.05 versus control group and ^∗∗^*P* < 0.05 versus LPS-treated group).

In this preliminarily study, we investigated the cytotoxic nature of DML in BV-2 microglial cells, to observe the toxic traits of the extract. The cells were treated with various concentrations of DML (100, 250, and 500 *μ*g/ml) alone or with LPS (200 ng/ml), and the cytotoxicity was assayed using MTT. The results of our study indicated that at selected concentrations, LPS alone or with DML-treated cells did not show any significant toxicity. Interestingly, we observed that the DML alone at the chosen higher concentration of 500 *μ*g/ml showed no toxic effects in BV-2 cells (Figure 1(a)). To further evaluate the inhibitory effect of DML in LPS-induced inflammatory responses, NO released from the DML-pretreated (100, 250, and 500 *μ*g/ml) LPS-inflicted cells or DML-treated cells alone was analyzed. Cells treated with DML alone did not exhibit any changes in NO levels, similar to the control cells. On the other hand, cells incubated with LPS (200 ng/ml) significantly elevated the NO release (28 ± 2.2 *μ*M, *P* < 0.05), which was dose dependently suppressed by DML treatment at described doses with values of 16.5 ± 0.3 *μ*M, 11.7 ± 0.2 *μ*M, and 4.9 ± 0.7 *μ*M, respectively (Figure 1(b)).

### 3.2. DML Attenuates the Proinflammatory Mediators in LPS-Stimulated BV-2 Cells

To examine the impact of DML in modulating the proinflammatory mediators, the BV-2 microglial cells were stimulated with LPS (200 ng/ml) and treated with or without DML at indicated concentrations (100, 250, and 500 *μ*g/ml). The alterations in the mRNA levels of the proinflammatory cytokines (TNF-*α*, IL-1*β*, and IL-6) and associated mediator (iNOS, COX-2) genes were observed after 6 h followed by LPS induction, using RT-PCR analysis. The densitometric analysis of the bands showed that LPS-stimulated BV-2 cells significantly upregulated the proinflammatory cytokine level with a ninefold increase in TNF-*α*, sixfold increase in both IL-1*β* and IL-6 level, and around fourfold increase in both iNOS and COX-2 level compared to the control group. Meanwhile, pretreatment of LPS-stimulated BV-2 cells with DML for 1 h effectively alleviated the upregulation of proinflammatory cytokines (TNF-*α*, IL-1*β*, and IL-6) and associated mediator (iNOS, COX-2) (Figures 1(c)–1(h)) genes in a dose-dependent manner. These results indicate that DML substantially suppress the proinflammatory mediators at the transcriptional level.

### 3.3. DML Attenuates the Inflammatory Protein Expression Level in LPS-Stimulated BV-2 Cells

To evaluate the effect of DML on the inflammatory protein expression in LPS-stimulated BV-2 microglia, the cells were pretreated with DML at described concentrations (100, 250, and 500 *μ*g/ml) and after 1 h incubated with LPS (200 ng/ml) for 24 h, followed by an evaluation of the protein markers using a Western blot analysis. The LPS-induced BV-2 cells significantly upregulated the iNOS and COX-2 protein levels with around a onefold increase compared to the control groups, and these have been substantially alleviated by DML treatment in a dose-dependent manner (Figures 1(i) and 1(j)).

### 3.4. DML Inhibits Inflammatory Response in LPS-Stimulated BV-2 Microglial Cells via Regulation of NF-*κ*B and JNK Pathways

**Figure 2:**
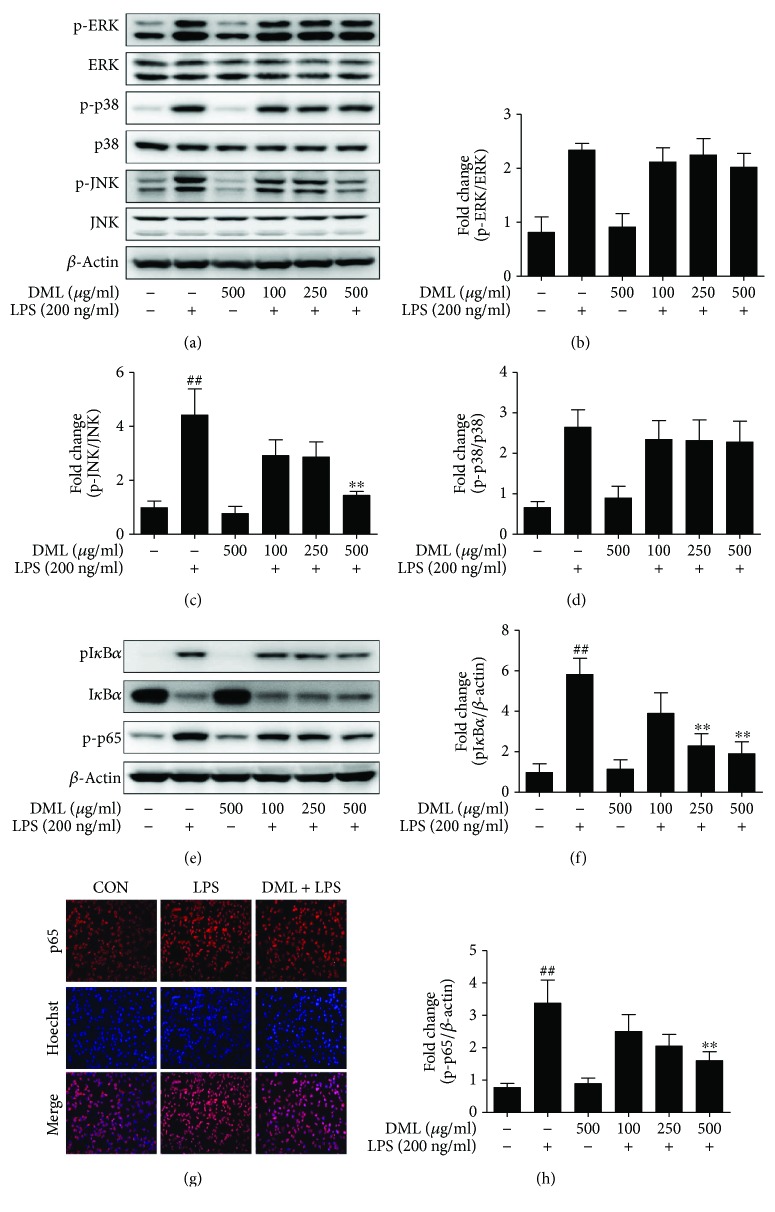
DML modulates the protein levels of MAPKs, NF-*κ*B, and I*κ*B-*α* in LPS-stimulated BV-2 cells. The protein expressions of MAPKs (a–d) were represented as blots and fold-change quantification with respect to their corresponding phosphorylated protein ratio. The I*κ*B-*α*, phospho-I*κ*B-*α*, and phospho-p65 (e, f, and h) were represented with respect to *β*-actin ratio. Data were mean ± standard error (*n* = 3) of three independent experiments. Values are mean ± standard error (^##^*P* < 0.05 versus control group and ^∗∗^*P* < 0.05 versus LPS-treated group). The subcellular location of NF-*κ*B p65 subunit was determined by immunofluorescence assay (g), using Alexa Fluor® 568 red with Hoechst background staining.

To explore the underlying molecular mechanism through which DML inhibits the inflammatory response in LPS-stimulated BV-2 microglia, we evaluated the protein expression level of the biomarkers associated with the NF-*κ*B pathway (p-I*κ*B-*α*, p-p65) and MAPK pathway biomarkers (p-ERK, p-p38, and p-JNK). BV-2 microglia cells were pretreated with DML at described concentrations (100, 250, and 500 *μ*g/ml) for one hour, incubated with LPS (200 ng/ml) for 30 min, and then evaluated for protein markers via Western blot. Our results indicate that the LPS stimulation significantly improved the I*κ*B-*α* phosphorylation, nuclear translocation, and phosphorylation of the NF-*κ*B p65 subunit and also involved in the upregulated phosphorylation of ERK, p38, and JNK protein expression in BV-2 cells. In contrast, the DML dose dependently suppressed the phosphorylation of both the I*κ*B-*α* and NF-*κ*B p65 subunit (Figures 2(e)–2(h)). Interestingly, among the evaluated MAPK biomarkers, DML suppressed only the phosphorylation of JNK and showed no effects for ERK and p38 (Figures 2(a)–2(d)). Thus, from this data, we understand that the DML effectively suppressed the inflammatory response in BV-2 cells by regulating both NF-*κ*B and JNK pathways.

### 3.5. DML Alleviated the Tyrosine Hydroxylase (TH) Depletion in MPTP-Intoxicated Mouse Model of PD

**Figure 3:**
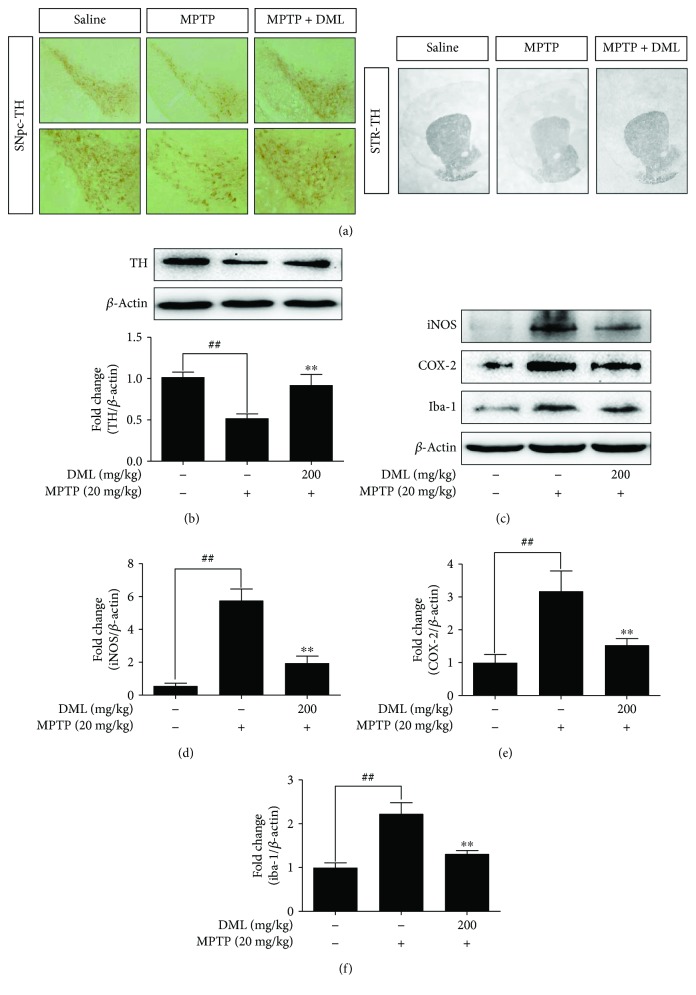
Defensive effect of DML on MPTP-induced loss of TH and elevated inflammatory mediator expression in substantia nigra pars compacta (SNpc). (a) Representative image of TH-positive cell immunoreactivity (IR) in substantia nigra pars compacta (SNpc) section and optical density (OD) analysis for TH-positive fibers in striatum (STR) section. The protein expressions of TH (b), iNOS, COX-2, and Iba-1 (c–f) in ventral midbrain (VM) were represented as blots and fold-change quantification with respect to *β*-actin ratio for three independent experiments. The values were mean ± standard error (^##^*P* < 0.05 versus control group and ^∗∗^*P* < 0.05 versus MPTP-treated group).

To evaluate the protective effects of DML against MPTP-inflicted TH depletion in mice, the protein expression of TH levels in the ventral midbrain (VM) was evaluated via Western blot (Figure 3(b)) and the immunoreactivity of the TH-positive cells in SNpc and STR was obtained through IHC (Figure 3(a)). As expected, MPTP intoxication significantly reduced the TH protein level expression in VM (*P* < 0.05), with a relative loss of TH-immunopositive fibers in striatum and SNpc compared to the control group, whereas DML-treated mice showed significant (*P* < 0.05) elevation in TH protein levels with substantial protection of TH-immunopositive fibers in striatum and SNpc as in the control group.

### 3.6. DML Suppress the Inflammatory Protein Expression and Microglial Activation Markers in MPTP-Intoxicated Mouse Model of PD

Neuronal inflammation mediated by microglial cell activation is a clinical hallmark of PD. To evaluate the neuroprotective effect of DML, mice were intoxicated with MPTP (20 mg/kg of bw, i.p.) followed by DML pretreatment (200 mg/kg of bw, p.o.) or saline treated or untreated (control), and the characteristic inflammatory (iNOS, COX-2) and microglial activation (Iba-1) protein expressions of the brain procured from respective groups were analyzed via Western blot (Figure 3(c)). MPTP-intoxicated mice substantially upregulated the expression of inflammatory proteins (iNOS: sixfold/COX-2: twofold) (Figures 3(d) and 3(e)) and microglial activation (Iba-1: onefold) protein (Figure 3(f)) compared to the control group. This upregulation of the characteristic protein expression was significantly suppressed by DML treatment, and it is quite equivalent to that of control.

### 3.7. DML Improved the Behavioral Deficits in MPTP-Intoxicated Mouse Model of PD

**Figure 4:**
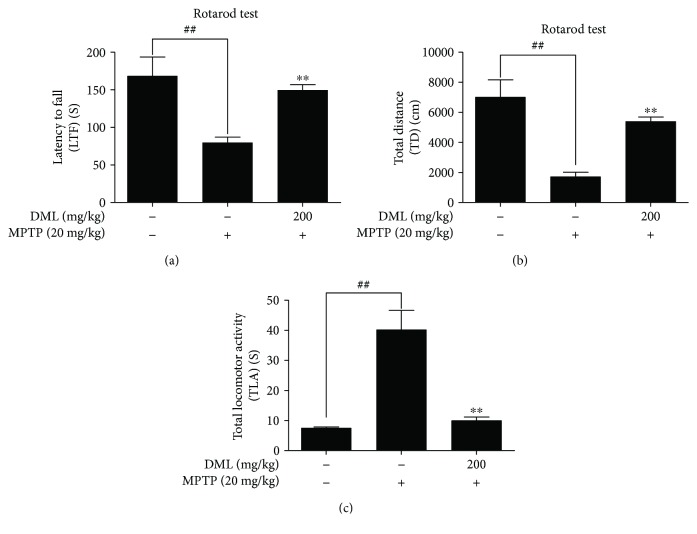
DML ameliorated the behavioral deficits in MPTP-intoxicated mouse. The rotarod and pole test were conducted on day 14. The latency of fall (LTF) time, total distance (TD), and total locomotor activity (TLA) of the animals were recorded and graphically represented in a, b, and c, respectively. Values shown were mean ± standard error for five mice (each group) (^##^*P* < 0.05 versus control group and ^∗∗^*P* < 0.05 versus MPTP-treated group).

To evaluate the protective effects of DML in terms of behavioral deficits, the mice were intoxicated with MPTP (20 mg/kg of bw, i.p.) followed by DML pretreatment (200 mg/kg of bw, p.o.) or saline treated or untreated (control), and their behavioral modulations were evaluated by performing a pole test and a rotarod test. The MPTP-intoxicated mice significantly reduced the latency to fall (LTF) (85.7 ± 20.9 s) (Figure 4(a)) and total distance (TD) covered (1.7 ± 0.7 m) (Figure 4(b)) in the rotarod test with an increased total locomotor activity (TLA) (45.4 ± 17.8 s) (Figure 4(c)) in the pole test, compared to the control group, whereas pretreatment with DML significantly ameliorated the behavioral deficit with improved LTF (139.7 ± 30.8 s) (Figure 4(a)), TD (4.3 ± 1.5 m) (Figure 4(b)), and reduced TLA (8.7 ± 3.1 s) (Figure 4(c)). This data suggests that DML improves the behavioral deficits exerted by MPTP intoxication in a mouse model of PD.

### 3.8. Chromatographic Analysis of DML/Fractions and Quantification of Its Phenolic Constituents

**Figure 5:**
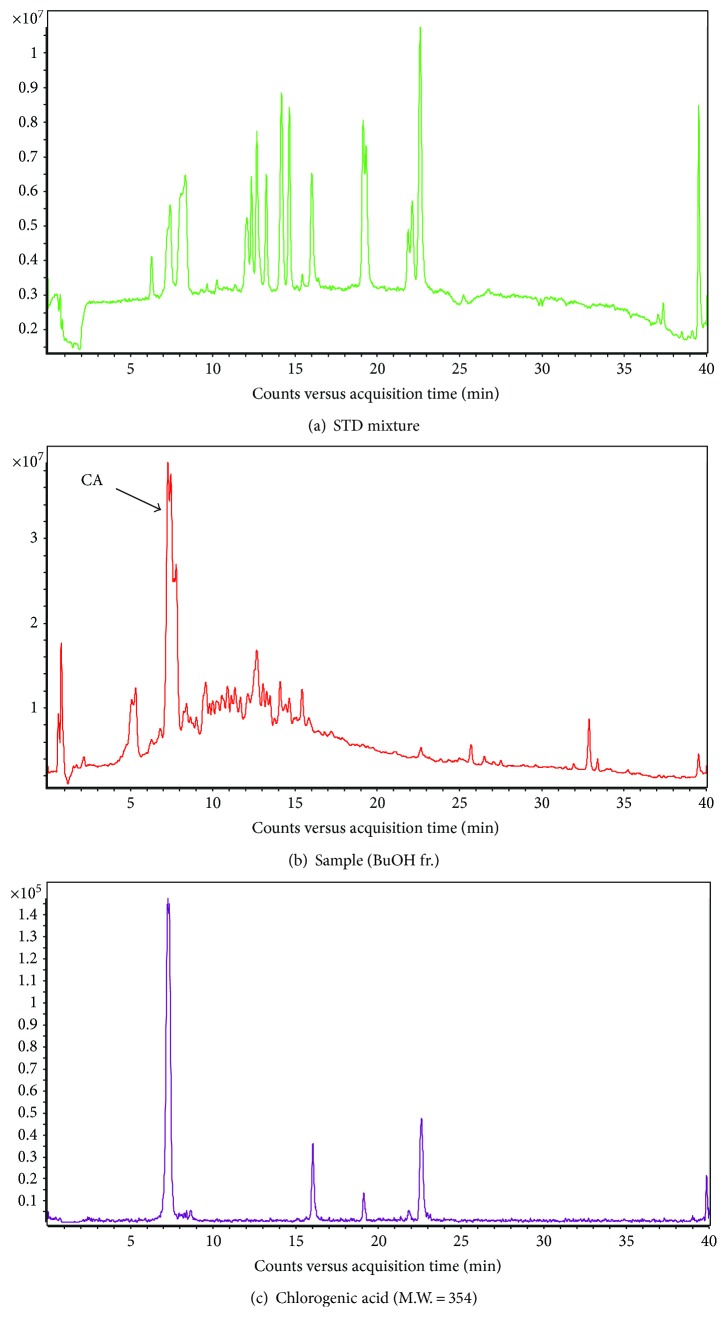
HPLC chromatograms of butanol fractions from aqueous extract of DML: (a) standard mixture, (b) butanol fraction of DML extract, and (c) chlorogenic acid standard.

Among the chromatographic profiles of DML and its fractions, the butanol fraction of DML exhibited a relatively higher quantity of phenolic constituents, which have been quantified and enlisted in Table 1. Despite this, the chlorogenic acid (CA) was reported to be present in a higher amount (19.5 mg/g of fraction) in the butanol fraction compared to other quantified phenolic constituents. The chromatographic profiles of the standard mixtures, DML-BuOH fraction, and CA are represented in Figures 5(a)–5(c), respectively.

### 3.9. Chlorogenic Acid Alleviates the Proinflammatory Mediators in LPS-Stimulated BV-2 Cells

**Figure 6:**
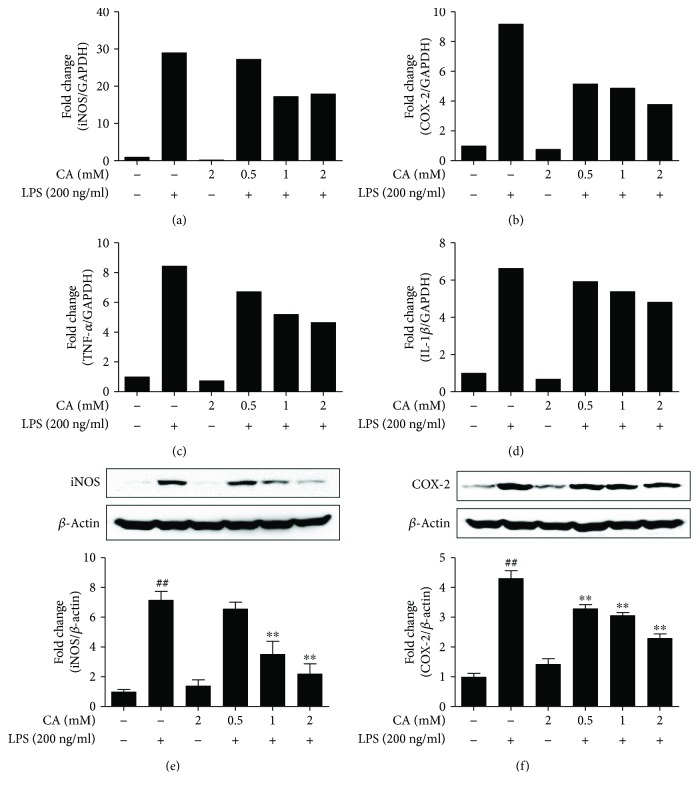
Modulatory effects of chlorogenic acid (CA) on proinflammatory cytokines and inflammatory mediators in LPS-activated BV-2 microglial cells. Cells were incubated with various concentrations of CA (0.5, 1, and 2 mM) in the presence or absence of LPS (200 ng/ml) for 6 h (RNA levels) and 24 h (protein levels). The qPCR results of the inflammatory cytokines were expressed as bands and fold-change quantification with respect to GAPDH ratio for iNOS (a), COX-2 (b), TNF-*α* (c), and IL-1*β* (d). The immunoblot results of inflammatory mediators iNOS (e) and COX-2 (f) were expressed as blots and fold-change quantification with respect to *β*-actin ratio. Data were mean ± standard error (*n* = 3) of three independent experiments. Values were mean ± standard error (^##^*P* < 0.05 versus control group and ^∗∗^*P* < 0.05 versus LPS-treated group).

In accordance with our chromatographic results, CA was found to be a major bioactive constituent of DML-butanol fraction. Thus, we further extended our study to evaluate the antineuroinflammatory potential of CA. The qRT-PCR and Western blot results of our study indicated that CA substantially downregulated the gene (Figures 6(a)–6(d)) as well as the protein expressions (Figures 6(e) and 6(f)) of proinflammatory cytokines and associated inflammatory mediators in a dose-dependent manner in LPS-induced BV-2 microglial cells. This result indicates that CA could possibly contribute to the antineuroinflammatory and ameliorative potential of DML in the MPTP-induced PD model.

## 4. Discussion

Neuroinflammation is a major hallmark of various progressive neurodegenerative disorders, including PD [29, 30]. An increase in *in vitro* and *in vivo* evidence suggests that inhibiting inflammatory cascades mediated by microglial activation is an effective therapeutic strategy to curb the progression of PD [31–34]. Lately, DML was also reported to show remedial effects on few neuronal-associated complications, that is, paralysis, stroke, and migraines [35]. Thus, in this study, we investigated the antineuroinflammatory and neuroprotective role of DML in the MPTP model. DML substantially curbed the advancement of microglia-mediated neuroinflammatory cascades both *in vitro* and *in vivo* and effectively alleviated the behavioral deficiencies detected in an MPTP-intoxicated PD mouse model. The resting microglia can be evoked by various inflammatory insults, such as LPS/MPTP-induced toxicity, which in turn leads to an upsurge in proinflammatory cytokines (TNF-*α*, IL-1*β*, and IL-6) and inflammatory mediators (COX-2 and inducible nitric oxide synthase (iNOS)), generating a ROS/RNS environment [14, 36, 37]. Jiang et al. described the antineuroinflammatory role of *Acorus gramineus* leaf aqueous extract by elucidating its potential modulating role on inflammatory mediators in MPTP-induced mouse model of Parkinson's disease [38]. A recent study reported that the aqueous extract of *Withania somnifera* leaves effectively suppressed the LPS-induced microglial inflammatory action by modulating the reported inflammatory mediators [39]. Accordingly, in our study, DML evidently alleviated the inflammatory effects inflicted by LPS- and MPTP-mediated glial cell activation both *in vitro* and *in vivo*, respectively, with a significant downregulation of proinflammatory cytokine (TNF-*α*, IL-1*β*, and IL-6) levels and inflammatory mediators (iNOS and COX-2). The underlying neuroinflammation event has been generally reported to be interlinked with several molecular pathways, among them are NF-*κ*B and MAPK signaling pathways, which are well documented [8, 40]. Phosphorylated I*κ*B-*α*-mediated nuclear translocation of NF-*κ*B and subsequent phosphorylation of mitogen-activated protein kinases (MAPKs, i.e., p-ERK, p-p38, and p-JNK) were reported to play an essential role in regulating transcriptional genes for iNOS and COX-2 production [40–42]. In this study, DML effectively suppressed the activation of NF-*κ*B by inhibiting the phosphorylation of I*κ*B-*α* and mediating the nuclear translocation of NF-*κ*B p65 subunit. Consequently, this inhibits iNOS and COX-2 production. Interestingly, our results also indicated that DML achieved the same results by suppressing the phosphorylation of JNK, but not other MAPKs. Earlier studies employed Iba-1 as an effective biomarker to positively stain the activated microglia, immunohistochemically [43]. In this study, the microgliosis inhibitory action of DML was double confirmed by capturing its suppressive role on microglial activation, with a decreased Iba-1 immunoreactivity in DML-treated MPTP-intoxicated mouse brains. Since DML inhibits neuroinflammation by targeting multiple molecular targets, it can be further developed as a potential therapeutic agent to halt the neuroinflammation in PD progression. MPTP intoxication inflicts motor deficits and bradykinesia in mice [44–46]. Ren et al. reported that dihydromyricetin, a natural flavonoid from *Ampelopsis grossedentata* sp., effectively alleviated motor impairments inflicted by MPTP-intoxicated PD mouse model, as observed in climbing pole and rotarod test results [47]. Accordingly, the results of our study indicated that MPTP intoxication exhibited severe behavioral deficits, including prolonged TLA in the pole test with reduced LTF and TD in the rotarod test. This was significantly alleviated by the DML pretreatment and remarkably improved the behavioral motor function. Numerous studies documented the reduction of dopamine levels associated with enormous loss of tyrosine hydroxylase- (TH-) positive fibers in the striatum and SNpc as a characteristic event of PD progression [44, 48–51]. In accordance, TH-positive (TH^+^) stains as a potential marker to immunohistochemically depict the dopaminergic neuron status [52, 53]. In this study, the MPTP intoxication inflicted substantial DA neuronal loss at STR and SNpc, with a decline in TH staining. In contrast, DML-treated animals showed significant positive TH staining, which indicates the neuroprotective activity of DML by shielding the loss of DA neurons in the PD pathogenesis. The chromatographic profiling of the DML/fraction showed that the butanol fraction of DML exhibited a relatively higher concentration of phenolic-flavonoids. Previous reports indicated that phenolic-flavonoids, such as kaempferol, quercetin, apigenin, coumaric acid, and caffeic acid, were reported to possess anti-inflammatory potential. In accordance, in this study, among several identified phenolic constituents of DML-butanol fraction, CA was found to be present at a higher concentration, and it exhibited substantial antineuroinflammatory potential in LPS-induced microglia cells. It is also worth noting that CA has been earlier reported to have significant antioxidant, anti-inflammation, antidiabetic, anticarcinogenic, and antiobesity activities, thereby facilitating a nonpharmacological and noninvasive approach for treatment or prevention of some chronic disease [54]. Taken together, we report that CA is a potential therapeutic candidate that contributes to the enhanced neuroprotective potential of DML.

## 5. Conclusion

In conclusion, our study showed that DML significantly attenuated neuroinflammatory cascades in activated microglia and restored the behavioral motor deficits in PD progression. The underlying molecular mechanism of DML can be explained as it effectively curbs the microglia-stimulated neuroinflammation by modulating the NF-*κ*B/I*κ*B-*α* and JNK-MAPK signaling pathways. However, further preclinical/clinical investigations of DML/bioactive compound on the inflammation trail shall pave the way to produce an effective therapeutic candidate to treat various neurodegenerative disorders.

## Figures and Tables

**Table 1:** Quantification of phytophenolic constituents in DML-BuOH fraction.

Compounds	Contents (mg) (per 1 g aqueous DML)
Quercetin	0.1
Kaempferol	0.02
Rutin	6.38
Vitexin	0.23
Luteolin	0.02
Tricin	0.06
Ferulic acid	0.03
Chlorogenic acid	19.5
Caffeic acid	0.10
